# Wear resistance of three direct resin composites in artificial Saliva at varying pH levels

**DOI:** 10.3389/fdmed.2025.1694614

**Published:** 2025-11-03

**Authors:** Haibing Yang, Chen Xu, Lei He, Juzhong Tian

**Affiliations:** Department of Stomatology, The Changzhou No. 2 People’s Hospital, The Third Affiliated Hospital of Nanjing Medical University, Changzhou Medical Center of Nanjing Medical University, Changzhou, Jiangsu, China

**Keywords:** composite resin, wear, pH, artificial saliva, SEM

## Abstract

**Objective:**

To investigate the wear resistance of three resin composites in artificial saliva at varying pH levels.

**Methods:**

Three resin materials—Coltene BRILLIANT™NG, 3M ESPE™Filtek™P60, and Kerr Sonicfill™2—were selected and subjected to reciprocating friction tests in artificial saliva with pH values of 2, 6.8, and 8. Wear volume was measured using a three-dimensional profilometer, and statistical analysis was performed using two-way ANOVA to compare differences in material loss among the resin groups and natural tooth enamel, considering both material type and pH as factors. Surface morphology of worn samples was analyzed via SEM.

**Results:**

Wear scar analysis revealed no statistically significant differences in wear volume among groups under pH 6.8 artificial saliva. In pH 2 artificial saliva, Group A (P60 resin) exhibited the highest wear volume, while Group B (Kerr SonicFill resin) showed the lowest wear volume, closely resembling that of natural enamel. Under pH 8 conditions, Group A again demonstrated the highest wear volume, whereas Group C (Coltene resin) exhibited the lowest. Group B (Kerr SonicFill) displayed wear volumes comparable to natural enamel (Group D). P60 resin showed significantly greater wear volume in pH 2 and pH 8 compared to pH 6.8. Kerr SonicFill resin exhibited lower wear volume in pH 2 than in pH 6.8 and pH 8, with no significant difference between pH 6.8 and pH 8. Coltene resin displayed higher wear volume in pH 2 and pH 6.8 compared to pH 8, but no significant difference was observed between pH 2 and pH 6.8. Natural enamel showed significantly greater volume loss at pH 8 compared to pH 6.8.

**Conclusion:**

Under the tested *in vitro* conditions, Kerr SonicFill resin demonstrated wear behavior most comparable to natural enamel across varying pH environments, showing stable performance. This suggests it could be a suitable choice for dental restorations requiring durability under varying pH conditions, though direct extrapolation to clinical performance requires caution. The increased wear of natural enamel at alkaline pH was an interesting finding warranting further study.

## 1 Introduction

A wide range of dental restorative materials are available, including silver amalgam, glass ionomer, and composite resins. Among these, composite resin is the most commonly used in clinical practice due to its aesthetic appeal, ability to replicate natural tooth morphology, high wear resistance, and minimal removal of tooth structure (1, 2). First introduced in the 1960s, composite resin is a polymer-based material composed of an organic matrix, coupling agents, inorganic fillers, initiators, and inhibitors (3). The inorganic fillers in light-cured resins are uniformly dispersed within the resin matrix, where they participate in photochemical reactions that initiate the polymerization of resin monomers into polymer networks. Upon exposure to light from the curing unit, photo-initiators within the resin are activated. This activation, facilitated by specific wavelengths of light, triggers interactions with the initiators, leading to polymerization and the progressive curing of the material into a fully hardened light-cured resin.

In recent years, significant advancements have been made in the physical and mechanical properties of composite resins, driven by modifications in their composition, including the enhancement of fillers, organic matrices, and coupling agents (1). One of the most significant improvements in commercial composite resins is the progressive reduction in the size of reinforced fillers, leading to the development of nanohybrid and nanoparticle-filled resins. Studies have demonstrated that nanocomposites (nanohybrid or nanoparticle-filled composites) exhibit superior polishability and reduced wear compared to traditional microhybrid or hybrid composites (4–7). Wear resistance is a critical factor in determining the longevity and effectiveness of dental restorations, as an ideal restorative material should exhibit wear characteristics comparable to natural tooth tissue (8). The wear of composite resins is influenced by multiple factors, including tooth properties, material composition, cavity size, occlusal relationships, and the characteristics of opposing teeth (9). The oral environment is highly dynamic and complex, with daily exposure to foods of varying pH levels and temperatures, as well as enzymatic activity in saliva, which may accelerate the hydrolytic degradation of composite resins (10–12). The chemical stability of the resin matrix, particularly the susceptibility of ester linkages in common monomers like Bis-GMA and UDMA to hydrolysis under low pH conditions, is a well-documented factor affecting material degradation (11, 13). Conversely, alkaline environments can compromise the silane coupling agent that bonds the inorganic fillers to the organic matrix, leading to filler debonding and accelerated wear (14). Understanding how these pH-dependent degradation mechanisms interact with mechanical wear is crucial for predicting the clinical performance of restorative materials.

Therefore, this study aims to simulate the oral environment and evaluate the surface morphology and material loss of three different resin composites in artificial saliva with varying pH levels, with the goal of identifying the most suitable composite resin for dental restorations under these specific conditions. We hypothesized that the wear resistance of the tested composite resins would vary significantly under different pH conditions, and that one material would demonstrate wear behavior most similar to natural enamel across the pH range tested. The novelty of this study lies in the direct comparative assessment of wear between modern composites and natural enamel across a clinically relevant pH spectrum, alongside the unexpected investigation of pH effects on enamel itself.

## 2 Materials and methods

### 2.1 Materials and equipment

The three resin materials used in this study were Coltene BRILLIANT™NG (Coltene/Whaledent AG, Altstätten, Switzerland), 3M ESPE™Filtek™P60 (3M Oral Care, St. Paul, MN, USA), and Kerr Sonicfill™2 (Kerr Corp., Orange, CA, USA), with their basic properties summarized in Supplementary Table S1. Natural tooth enamel samples were obtained from the Department of Stomatology, The Changzhou No. 2 People's Hospital, following approval by the Institutional Ethics Committee [Approval Number: (2019)KY051-01]. The frictional counter-body was composed of talc porcelain (Haimen Tianbu High-Frequency Ceramic Factory, Haimen, China; Vickers Hardness ∼600 HV, Elastic Modulus ∼70 GPa), chosen as a standardized antagonist material for wear testing according to ISO/TS 14569-2. Additional materials included self-curing acrylic plastic (Vertex Rapid Simplified, Vertex-Dental B.V., Zeist, Netherlands), silicone rubber (DMG, Hamburg, Germany), polishing sandpaper (SiC paper, Struers, Cleveland, OH, USA), polishing kits (Enhance and PoGo kits, Dentsply Sirona, Charlotte, NC, USA for resins; Dialite HP polishing kit, Brasseler USA, Savannah, GA, USA for enamel), and artificial saliva (NobleRyder C8029, Beijing Noble Ryder Technology Co., Ltd., China) with pH values of 2, 6.8, and 8. The composition of the artificial saliva was: KCl (0.4 g/L), NaCl (0.4 g/L), CaCl_2_·2H_2_O (0.906 g/L), NaH_2_PO_4_·2H_2_O (0.690 g/L), Na_2_S·9H_2_O (0.005 g/L), urea (1 g/L). The wear testing apparatus used was a high-speed reciprocating friction wear test machine (Model MDW-02, Jinan Yihua Tribology Testing Technology Co., Ltd., Jinan, China), while surface morphology analysis was performed using a scanning electron microscope (SEM) (Inspect F50, FEI Co., Hillsboro, OR, USA). A three-dimensional optical profilometer (ContourGT-K1, Bruker Corp., Billerica, MA, USA; vertical resolution <0.1 nm, lateral resolution ∼0.5 μm) was used for wear volume measurements.

### 2.2 Methods

#### 2.2.1 Sample preparation and grouping

The saliva chamber used in the experiment was a custom-made acrylic container with dimensions of 2.5 cm × 2.5 cm × 1.5 cm, featuring a lid with precisely positioned holes to securely hold the sample holder pin and the counter-body arm. This design ensured consistent immersion of the contact area in artificial saliva and maintained stable alignment during the reciprocating motion. Samples for wear testing were prepared for each material (P60, SonicFill, Coltene Brilliant NG) and for natural enamel. For each material/enamel group (*n* = 10 per group), samples were further subdivided for testing at the three different pH levels (pH 2, 6.8, 8), resulting in *n* = 3–4 samples per material per pH condition.

Sample preparation for Resin Groups (A, B, C): A specified amount of composite resin was placed onto a clean glass slide and spread using a resin filling instrument to form a rectangular block measuring 1.5 cm × 1.2 cm × 1.0 cm. Each surface of the block was light-cured with an LED light-curing unit (Bluephase G4, Ivoclar Vivadent, Schaan, Liechtenstein; irradiance set to 1,200 mW/cm^2^, verified using a calibrated radiometer) for 40 s per surface. The cured resin blocks were then sequentially polished using 320#, 800#, and 1,200# grit SiC sandpaper under running water using an automatic polisher (EcoMet 30, Buehler, Lake Bluff, IL, USA) at 150 rpm for 60 s per grit step to ensure a flat surface. This was followed by final polishing with a resin polishing kit (Enhance points for 60 s at 150 rpm, then PoGo points for 60 s at 150 rpm, Dentsply Sirona) to achieve a clinically relevant surface finish. The initial average surface roughness (Ra) for all polished resin samples was measured using the profilometer and was less than 0.05 µm.

Natural Tooth Enamel Sample Preparation (Group D): Ten intact human upper third molars, extracted for therapeutic reasons within the past two weeks from patients aged 18–30 years, were collected. Only teeth without occlusal contact, caries, demineralization, or developmental defects were selected. Ethical approval was obtained for the use of these teeth. After extraction, the teeth were stored in physiological saline at 4°C. The roots were sectioned off using a diamond saw under water irrigation. The crowns were then mounted in self-curing acrylic resin. The buccal enamel surface was chosen as the test area. This surface was ground flat using a turbine handpiece with water spray and sequentially polished with 320#, 800#, and 1,200# grit SiC sandpaper manually under running water to create a flat surface of approximately 4 mm × 4 mm, followed by final polishing with a Dialite HP polishing kit (Brasseler USA) using a slow-speed handpiece to achieve a smooth surface (Ra < 0.1 µm). The defined “appropriate size” for the test surface was a minimum exposed flat area of 2 mm×4 mm to accommodate the 4 mm wear track.

For both resin and enamel samples, approximately 2 mm height of the prepared test surface was exposed above the holding material. The remaining portion of each sample was embedded in DMG silicone rubber within a mold, ensuring a secure fit within the saliva chamber during testing. All samples were stored in distilled water at 37°C for 24 h prior to wear testing to allow for water saturation.

The counter-body was made of talc porcelain, with the contact end shaped into spherical cylinders (3 mm in diameter). A total of 40 counter-bodies were prepared. Prior to testing, all samples and counter-bodies were cleaned using a KS-500E ultrasonic cleaner (Kunshan Hechuang Ultrasonic Instrument Co., Ltd., China) in distilled water for 10 min, followed by three alternating washes (3 min each) in anhydrous ethanol and acetone. The samples were then air-dried with cold air before being used in the experiment.

#### 2.2.2 Friction and wear testing

**Figure 1:**
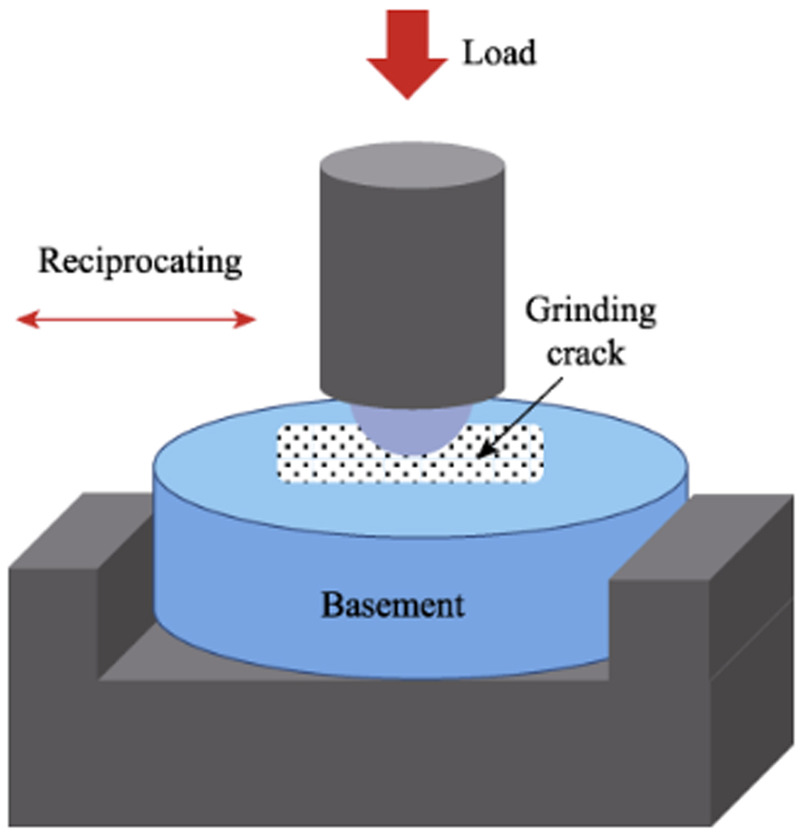
Schematic diagram of the reciprocating wear test setup.

A multi-functional surface performance testing instrument (Model MDW-02, Jinan Yihua Tribology Testing Technology Co., Ltd.) was used to perform a pin-on-disc reciprocating friction test. The setup consisted of the sample (disc) fixed in the chamber filled with artificial saliva, and the talc porcelain counter-body (pin) attached to the loading arm moving reciprocally over the sample surface (Figure 1). The normal chewing force in human dentition ranges from 3 to 36 N (15), with a typical sliding distance of 2–4 mm between the upper and lower teeth (4). The test parameters were set as follows: a vertical load of 20 N (within the typical masticatory force range), an operation time of 30 min (selected to generate measurable wear based on preliminary tests and equivalent to approximately 54,000 cycles at the chosen frequency, simulating a significant period of chewing activity), a reciprocating frequency of 30 Hz (resulting in 108,000 total cycles), and a sliding distance of 4 mm. The pH of the artificial saliva was adjusted to the target values (2.0, 6.8, 8.0) using 1 M HCl or 1 M NaOH solutions and was monitored before and after the test to ensure stability (±0.2 pH unit change). The wear resistance of the three resin materials and natural tooth enamel was evaluated using talc porcelain as the counter-body in artificial saliva at different pH values (2, 6.8, 8) under room temperature conditions (23 ± 2°C). The wear assessment adhered to ISO/TS 14569 standards, utilizing a profilometric method to simulate the masticatory process (16). The 4 mm sliding distance occurred entirely within the 2 mm exposed height of the sample surface; the DMG silicone rubber embedding material was not contacted by the counter-body during the test.

#### 2.2.3 Morphological analysis of worn surfaces

The worn sample surfaces were gold-sputter coated (Q150R ES, Quorum Technologies, Laughton, UK) and examined using a scanning electron microscope (SEM) (Inspect F50, FEI Co.) at an accelerating voltage of 10 kV and a working distance of approximately 10 mm. Images were taken at 500× and 3,000× magnifications to assess morphological changes in each group. Areas showing representative wear features (e.g., striations, pits, cracks) were selected for higher magnification imaging, avoiding areas predominantly containing debris.

#### 2.2.4 Measurement of wear volume

After ultrasonic cleaning (as in 1.2.1) and drying, the samples were scanned using the three-dimensional optical profilometer (ContourGT-K1, Bruker Corp.), which was calibrated prior to measurements using a standard reference artifact. The cross-sectional profile of the wear scar was extracted perpendicular to the sliding direction at three different locations along the track (start, middle, and end). The average cross-sectional area of the wear pit was calculated using the instrument's software (Vision64, Bruker Corp.) by first defining a reference plane based on the unworn surface areas adjacent to the scar. The software then calculated the area of the material missing below this reference plane (negative volume) for each profile using numerical integration. The wear volume was then determined by multiplying the average cross-sectional area by the total sliding distance of 4 mm. The repeatability of the volume measurement was high, with a coefficient of variation of less than 5% for repeated scans of the same wear scar. This measurement pertained only to the wear of the sample material (resin or enamel), not the silicone rubber.

#### 2.2.5 Statistical analysis

Statistical analysis of wear volume among groups was performed using GraphPad Prism 6 (GraphPad Software, Inc., La Jolla, CA, USA) [Citation: GraphPad Prism, Version 6, GraphPad Software, Inc., La Jolla, CA, USA]. Data were analyzed using a two-way analysis of variance (ANOVA) to assess the effects of the two independent variables (material type and pH level) and their interaction on wear volume. This was followed by Tukey's honestly significant difference (HSD) *post-hoc* test for multiple comparisons. Statistical significance was set at *P* < 0.01. Data are presented as mean ± standard deviation (SD).

## 3 Results

### 3.1 Volume loss Due to wear

The mean wear volumes and standard deviations for all material groups at each pH level are summarized in Supplementary Table S2.

**Figure 2:**
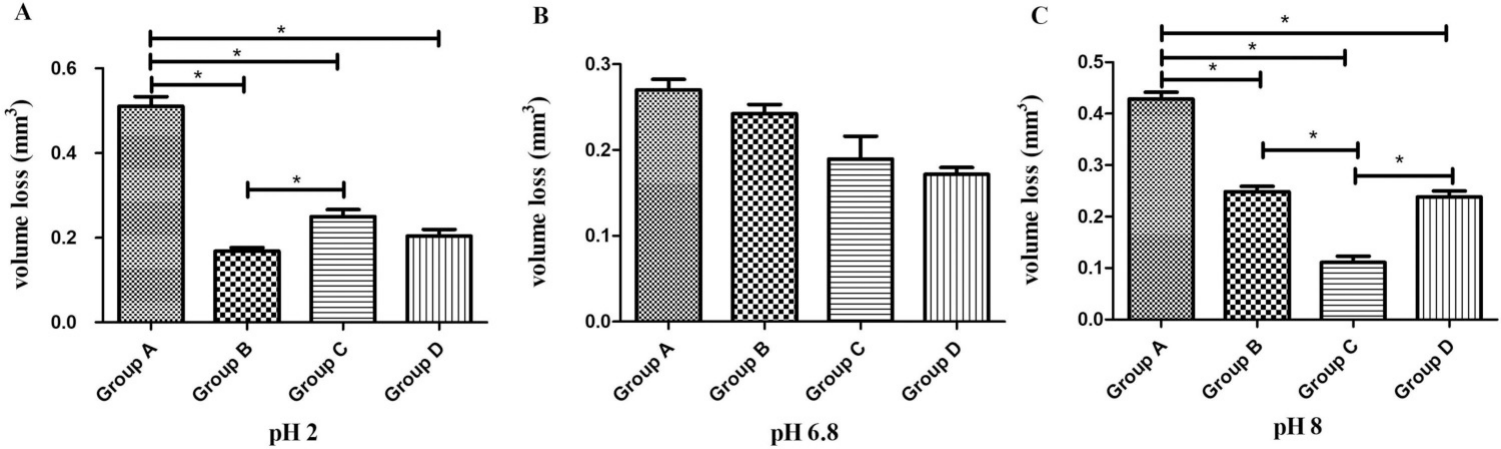
Comparison of wear volume (mm^3^, mean ± SD) among four groups of materials at three different pH levels. **(A)** Wear volume at pH 2. **(B)** Wear volume at pH 6.8. **(C)** Wear volume at pH 8. * indicates significant difference (*P* < 0.01).

Under pH 2 artificial saliva (Figure 2A), Group A (P60) exhibited the highest volume loss, showing statistically significant differences compared to all other groups (*P* < 0.01, Tukey's HSD). Group B (Kerr SonicFill) had the lowest volume loss, with significant differences compared to Groups A and C (*P* < 0.01) but no significant difference compared to Group D (Natural enamel) (*P* > 0.01).

Under pH 6.8 artificial saliva (Figure 2B), no statistically significant differences in volume loss were observed among the groups (*P* > 0.01, Tukey's HSD), indicating comparable wear resistance under neutral conditions.

Under pH 8 artificial saliva (Figure 2C), Group A (P60) again showed the highest volume loss, with statistically significant differences compared to all other groups (*P* < 0.01, Tukey's HSD). Group C (Coltene) exhibited the lowest volume loss, significantly differing from all other groups (*P* < 0.01). No statistically significant difference was observed between Groups B (Kerr SonicFill) and D (Natural enamel) (*P* > 0.01).

**Figure 3:**
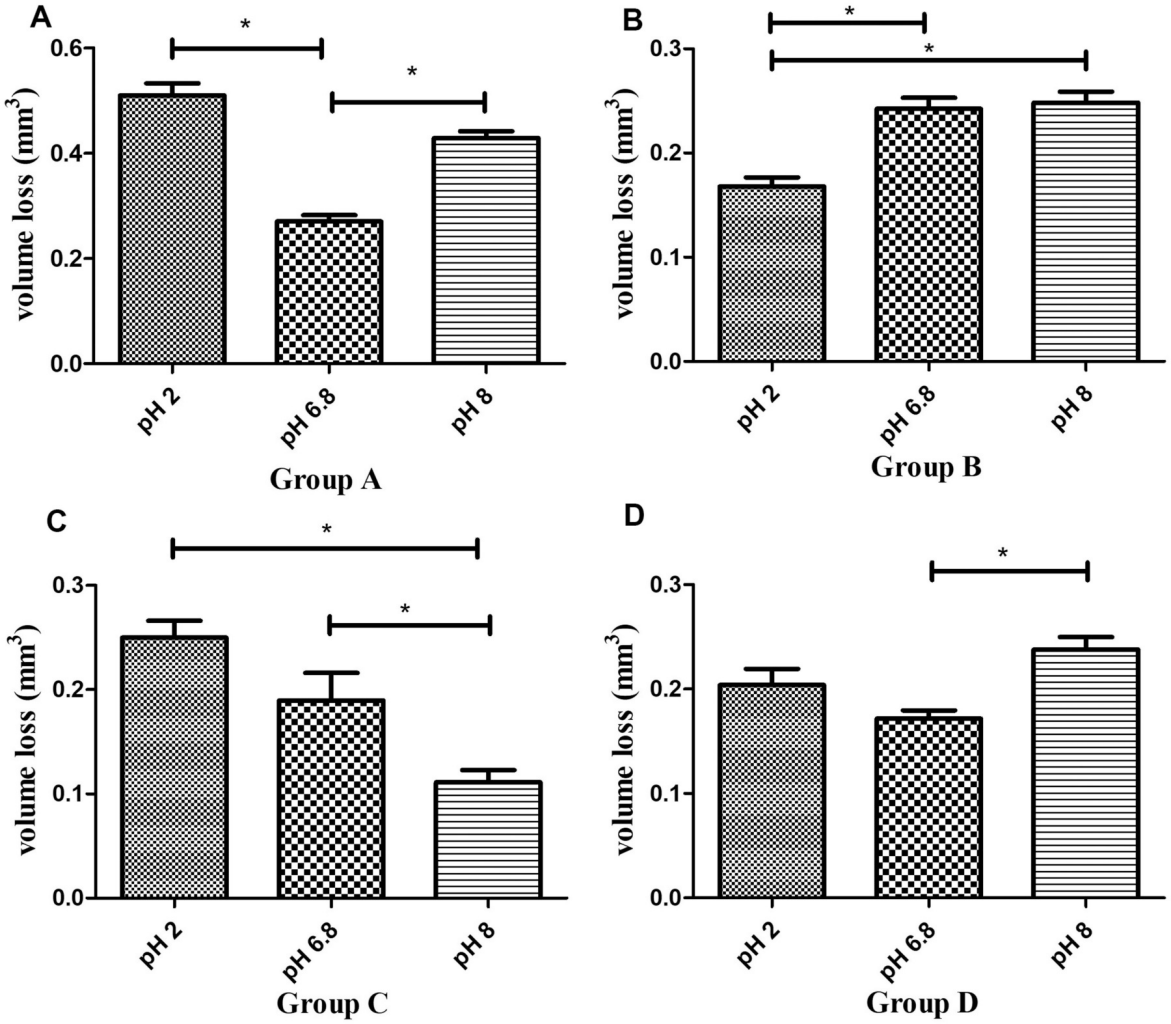
Comparison of wear volume (mm^3^, mean ± SD) of four groups of materials across different pH levels. **(A)** Wear volume of P60 resin composite at pH 2, 6.8, and 8. **(B)** Wear volume of Sonicfill™2 resin composite at pH 2, 6.8, and 8. **(C)** Wear volume of Brilliant NG resin composite at pH 2, 6.8, and 8. **(D)** Wear volume of natural enamel at pH 2, 6.8, and 8. * indicates significant difference (*P* < 0.01).

Post-hoc comparisons within each material across pH levels (Figure 3) showed:

- P60 resin (Figure 3A) exhibited significantly greater volume loss in pH 2 and pH 8 compared to pH 6.8 (*P* < 0.01, Tukey's HSD).
- Kerr SonicFill resin (Figure 3B) displayed significantly lower volume loss in pH 2 compared to pH 6.8 and pH 8 (*P* < 0.01), while no significant difference was observed between pH 6.8 and pH 8 (*P* > 0.01).
- Coltene resin (Figure 3C) exhibited significantly greater volume loss in pH 2 and pH 6.8 compared to pH 8 (*P* < 0.01), but no significant difference was observed between pH 2 and pH 6.8 (*P* > 0.01).
- Natural tooth enamel (Figure 3D) showed significantly greater volume loss in pH 8 compared to pH 6.8 (*P* < 0.01), whereas no significant difference was found between pH 2 and pH 6.8 (*P* > 0.01). The increased wear of enamel at alkaline pH was a surprising result.

These findings indicate that P60 resin is more vulnerable to both acidic and mildly alkaline conditions, while Kerr SonicFill resin exhibits enhanced resistance to acidic wear. Coltene resin demonstrates superior wear resistance in mildly alkaline conditions, whereas natural enamel shows increased wear in high-pH environments.

### 3.2 Morphology of worn surfaces

**Figure 4:**
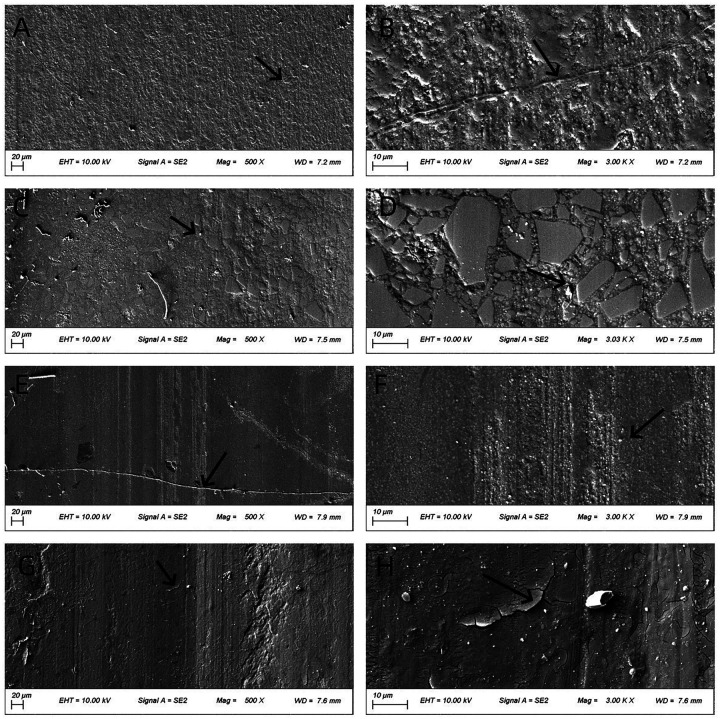
SEM images (500× and 3,000×) of worn surfaces after testing in artificial saliva (pH 2). **(A,B)** P60; **(C,D)** Kerr SonicFill; **(E,F)** Coltene Brilliant NG; **(G,H)** natural enamel. (Arrows indicate key features: grooves in **(A,B)**, pits/filler detachment in **(C–F)**, striations and cracks in **(G,H)**).

**Figure 5:**
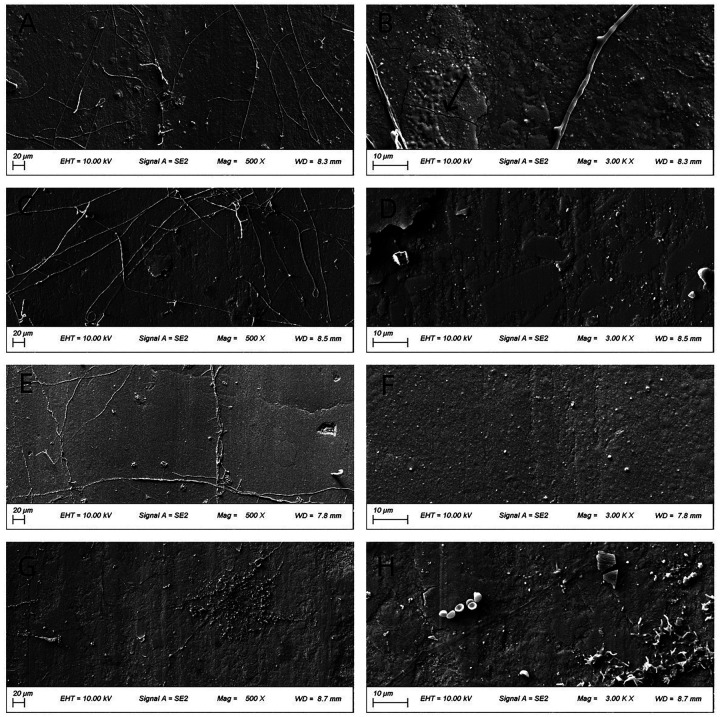
SEM images (500× and 3,000×) of worn surfaces after testing in artificial saliva (pH 6.8). **(A,B)**: P60; **(C,D)** Kerr SonicFill; **(E,F)** Coltene Brilliant NG; **(G,H)** natural enamel. [Arrows indicate key features: grooves in **(B)**].

**Figure 6:**
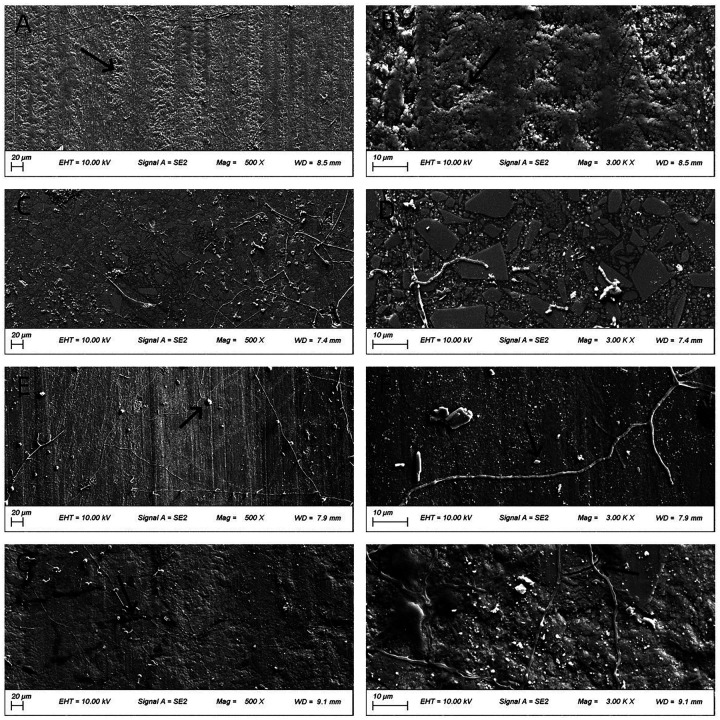
SEM images (500× and 3,000×) of worn surfaces after testing in artificial saliva (pH 8). **(A,B)** P60; **(C,D)** Kerr SonicFill; **(E,F)** Coltene Brilliant NG; **(G,H)** natural enamel. [Arrows indicate key features: wear track in **(A,B)**, pits/filler detachment in **(E,F)**, striations and cracks in **(G,H)**)].

Figures 4–6 present low- (500×) and high-magnification (3,000×) SEM images of the worn surfaces of each material following wear testing in artificial saliva at pH 2, 6.8, and 8, respectively. The images correspond to: (A) P60 (low magnification); (B) P60 (high magnification); (C) Kerr Sonicfill (low magnification); (D) Kerr Sonicfill (high magnification); (E) Coltene (low magnification); (F) Coltene (high magnification); (G) Natural enamel (low magnification); (H) Natural enamel (high magnification). Some debris is visible on the enamel surfaces, likely remnants from the wear process or preparation; however, the images focus on areas demonstrating characteristic wear features.

Figure 4 illustrates the worn surface morphology of all groups after wear testing in artificial saliva (pH 2). The P60 resin exhibited a relatively dense but ploughed surface with evident grooves. The Coltene resin and natural tooth enamel showed smoother surfaces with distinct frictional striations. The Coltene resin displayed exfoliative pits between the striations, whereas natural enamel showed enamel cracks with unclear enamel prism cross-sections. The Kerr SonicFill resin exhibited some pitting and voids due to filler particle detachment.

Figure 5 illustrates the worn surface morphology of all groups following wear testing in artificial saliva (pH 6.8). Compared to pH 2 conditions, the frictional striations were less pronounced across all groups. The overall surface integrity of the materials appeared better preserved. Fewer pits and cracks were observed compared to the acidic environment.

Figure 6 presents the worn surface morphology of the four groups after wear testing in artificial saliva (pH 8). P60, Coltene, and natural enamel exhibited more pronounced frictional striations than those observed at pH 6.8. Coltene resin displayed fewer pits compared to its morphology at pH 2. Kerr SonicFill showed no significant morphological changes compared to other pH conditions. Natural enamel showed pronounced striations and some micro-cracking.

## 4 Discussion

This study evaluated the wear resistance of three commercial composite resins and natural tooth enamel under different pH conditions simulating the oral environment. The results confirmed our hypothesis that wear resistance is significantly influenced by pH, and the extent of this effect depends on the material composition. The finding that Kerr SonicFill's wear was closest to enamel across pH variations, coupled with the unexpected susceptibility of enamel itself to higher wear at pH 8, are the key novel contributions of this work.

Mastication is a highly dynamic and complex process, subjecting dental restorative materials to continuous mechanical forces, temperature fluctuations, humidity variations, and pH changes, all of which contribute to material aging and alterations in wear resistance (9–11). The observed differences in wear volume and surface morphology under varying pH conditions can be attributed to chemical degradation mechanisms potentially superimposed on mechanical wear. Acidic environments (pH 2) likely promote hydrolysis of the resin matrix, particularly ester linkages in Bis-GMA, UDMA, and other monomers (11–13, 17). This hydrolysis weakens the matrix, compromises the filler-matrix interface (often mediated by silane coupling agents), and facilitates filler dislodgement, leading to increased wear (10, 15). The extent of hydrolytic degradation depends on the specific monomer composition and the cross-linking density of the polymer network (13, 18). Materials with a higher concentration of hydrolytically stable monomers or a denser network may exhibit better resistance. This mechanism is consistent with the increased wear volume observed for P60 and Coltene resins at pH 2 (compared to neutral pH) and the corresponding SEM observations of pits and voids. The superior performance of SonicFill at low pH might be related to its specific proprietary modified Bis-GMA/TEGDMA matrix composition, which may offer greater hydrolytic stability, its high nano-hybrid filler load (84% wt), and potentially more stable silane coupling, offering better overall resistance to hydrolytic degradation and filler loss (14, 19). Furthermore, the sole influence of pH on composite surface properties, such as causing microcracks or increasing surface roughness even before mechanical testing, has been reported (18). Although not directly measured in this study pre-wear, such pre-existing damage could predispose the material to higher wear rates.

The mildly alkaline environment (pH 8) might affect the silane coupling agent (often susceptible to degradation under high pH), potentially leading to filler debonding (14). This could explain the increased wear observed for P60, which has a lower filler load (61% by weight, corresponding to a lower volume fraction), at high pH. The excellent wear resistance of Coltene Brilliant NG resin at pH 8, even showing lower volume loss than natural enamel, might be due to its specific filler technology (combination of nano-particles and pre-polymerized particles) and matrix composition (Bis-GMA, UDMA, TEGDMA) that remains stable or may even benefit from the mildly alkaline conditions. The relatively stable urethane linkages in UDMA might contribute to this alkaline resistance compared to ester-rich matrices. However, this high resistance raises a clinical consideration: if a restorative material wears significantly less than enamel, it might cause excessive wear of the opposing natural tooth over time (8). Therefore, while Coltene Brilliant NG demonstrated superior wear resistance at pH 8, its clinical recommendation should be made with caution, considering the potential for antagonist tooth wear. The goal is a material with wear behavior closely matched to enamel, not one that is excessively more resistant.

The most striking finding regarding natural enamel was its significantly higher wear at pH 8 compared to pH 6.8 and pH 2. While enamel is known to demineralize in acid, its relative susceptibility to wear in mildly alkaline conditions is less commonly reported and warrants further investigation. It might be related to changes in the organic component (e.g., degradation of enamel proteins) or the hydration layer of enamel under alkaline conditions, potentially altering its tribological properties and making it more susceptible to mechanical abrasion (20). Alternatively, the chemical interaction at pH 8 might affect the carbonate or phosphate ions in hydroxyapatite, slightly reducing its mechanical resilience. This finding aligns with some studies suggesting alterations in enamel surface energy or hardness in non-acidic environments (21).

The SEM observations generally correlated with the volumetric wear data. The pronounced striations and cracks seen on enamel and some resins at extreme pH values support the increased material loss measured profilometrically. The stability of Kerr SonicFill's morphology across pH conditions aligns with its relatively stable wear performance, particularly its resistance to acid.

Kerr SonicFill resin incorporates nano-fillers, including glass fibers and silica, with a high filler content (84% wt, implying a high volume fraction) and reportedly stable matrix chemistry (14, 19). According to the abrasive wear mechanism, a higher filler content combined with smaller particle sizes reduces the inter-filler gaps, thereby preventing abrasive particles from penetrating and degrading the resin matrix (4, 22). This structural integrity enhances filler retention within the matrix, minimizing their likelihood of detachment. Furthermore, smaller filler particles contribute to lower volume loss upon detachment, leading to superior overall wear resistance (1, 4). Coltene “Brilliant” resin combines nano-particles and pre-polymerized particles, which might contribute to its performance under alkaline conditions. FiltekTM P60 resin, a microhybrid composite with a slightly lower filler volume ratio (61% by weight, corresponding to a lower volume fraction than nanohybrids), might be more susceptible to matrix degradation and filler loss under chemical challenge (19, 23). The use of weight percentage (wt%) for SonicFill and volume fraction implied from weight percentage for P60 in the discussion is due to the common reporting practices in the respective material datasheets and literature. However, volume fraction is often considered a more direct predictor of mechanical properties (1).

Our findings agree with previous studies highlighting the importance of filler content and composition on wear resistance (4, 19, 22, 23). Studies have also shown that pH influences resin degradation (11–13, 18, 24), but direct comparisons are complex due to variations in experimental methods. The use of a standardized wear tester with a low coefficient of variation (<5%) (25) enhances the reliability of our comparative results.

This study has limitations. It is an *in vitro* simulation using a ceramic counter-body, which cannot fully replicate the complexity of the oral environment, including the presence of enzymes, biofilms, and the exact nature of antagonistic tooth contact. Additionally, the study did not assess surface properties like gloss or roughness before and after wear, which are important clinical indicators of surface stability (26). The initial surface roughness was controlled (Ra < 0.05 µm for resins), but post-wear gloss changes were not quantified. The conclusion that SonicFill's performance was most comparable to enamel is based on these specific test conditions and should be interpreted cautiously regarding clinical performance. Future studies should include long-term aging (e.g., thermocycling), chemical analysis of degradation products, testing against human enamel cusps, further investigation of the unexpected enamel wear at high pH, and measurement of additional parameters like surface gloss to comprehensively assess surface changes.

Within the limitations of this *in vitro* study, the wear resistance of the tested composite resins was significantly influenced by the pH of the surrounding environment. Kerr SonicFill resin demonstrated the most consistent wear behavior across the pH range, with values closest to those of natural enamel under acidic and neutral conditions and comparable under mildly alkaline conditions. Filtek P60 resin showed the highest susceptibility to pH variations. Coltene Brilliant NG exhibited low wear under mildly alkaline conditions. The finding that natural enamel wear increased under mildly alkaline conditions requires further investigation. Based on these *in vitro* results, Kerr SonicFill appears to be a promising material for restorations exposed to varying pH environments due to its stable wear performance. Future research should focus on long-term clinical evaluations, understanding the chemical mechanisms of pH-induced wear, and exploring the wear behavior of enamel under alkaline conditions.
