# Supplementary material for: Wear resistance of three direct resin composites in artificial Saliva at varying pH levels
Source: Front Dent Med. 2025 Nov 3;6:1694614. doi: 10.3389/fdmed.2025.1694614 (PMC12620477; doi:10.3389/fdmed.2025.1694614)
Supplement: Supplementary file 1 [file Table1.docx]

| Material (Group) | Type | Matrix Composition | Filler Size | Filler Content(wt%) | Manufacturer |
| --- | --- | --- | --- | --- | --- |
| Filtek^TM^ P60 (A) | Microhybrid | Bis-GMA, UDMA, Bis-EMA | 0.1 - 3.0 µm | 61% | 3M ESPE, USA |
| Sonicfill^TM^2 (B) | Nanohybrid | Proprietary Modified Bis-GMA, TEGDMA | 20 nm - 5 µm | 84% | Kerr Corp. USA |
| BRILLIANT^TM^NG (C) | Nanohybrid | Bis-GMA, UDMA, TEGDMA | 40 nm - 3 µm | 78% | Coltene, Switzerland |

Table 1. Basic properties of the tested resin composites.
