# Supplementary material for: Wear resistance of three direct resin composites in artificial Saliva at varying pH levels
Source: Front Dent Med. 2025 Nov 3;6:1694614. doi: 10.3389/fdmed.2025.1694614 (PMC12620477; doi:10.3389/fdmed.2025.1694614)
Supplement: Supplementary file 2 [file Table2.docx]

Table 2. Mean wear volume (mm³, mean ± SD) of tested materials in artificial saliva at different pH levels.

| Material (Group) | pH 2.0 | pH 6.8 | pH 8.0 |
| --- | --- | --- | --- |
| Filtek^TM^ P60 (A) | 0.51 ± 0.07163 | 0.2701 ± 0.03871 | 0.4287 ± 0.04103 |
| Sonicfill^TM^2 (B) | 0.1679 ± 0.02806 | 0.2425 ± 0.03347 | 0.2485 ± 0.03312 |
| BRILLIANT^TM^NG (C) | 0.2498 ± 0.05165 | 0.1896 ± 0.08357 | 0.1111 ± 0.03757 |
| Natural Enamel (D) | 0.2038 ± 0.04963 | 0.1717 ± 0.02458 | 0.238 ± 0.03719 |
